# Postoperative recovery patterns following discectomy surgery in patients with lumbar radiculopathy

**DOI:** 10.1038/s41598-022-15169-8

**Published:** 2022-07-01

**Authors:** Shuaijin Wang, Jeffrey J. Hebert, Edward Abraham, Amanda Vandewint, Erin Bigney, Eden Richardson, Dana El-Mughayyar, Najmedden Attabib, Niels Wedderkopp, Stephen Kingwell, Alex Soroceanu, M. H. Weber, Hamilton Hall, Joel Finkelstein, Christopher S. Bailey, Kenneth Thomas, Andrew Nataraj, Jerome Paquet, Michael G. Johnson, Charles Fisher, Y. Raja Rampersaud, Nicolas Dea, Chris Small, Neil Manson

**Affiliations:** 1 Faculty of Medicine, Dalhousie Medicine New Brunswick, Saint John, New Brunswick Canada; 2 Faculty of Kinesiology, University of New Brunswick, Fredericton, New Brunswick Canada; 3 Canada East Spine Centre, Saint John, New Brunswick Canada; 4 Saint John Orthopaedics, Saint John, New Brunswick Canada; 5 Horizon Health Network, Saint John, New Brunswick Canada; 6 Department of Regional Health Research, University of Southern Denmark, Odense, Denmark; 7 The Orthopedic Department, Hospital of Southwestern Jutland, Esbjerg, Denmark; 8 Canadian Spine Outcomes and Research Network, Markdale, ON Canada; 9 The Ottawa Hospital-Civic Campus, Ottawa, ON Canada; 10 Department of Surgery, University of Calgary, Calgary, AB Canada; 11 McGill University, Montreal, QC Canada; 12 Department of Surgery, University of Toronto, Toronto, ON Canada; 13 Division of Orthopedics and Spine Surgery, Sunnybrook Health Sciences Centre, Toronto, ON Canada; 14 London Health Science Centre, Western University, London, ON Canada; 15 Department of Surgery, Division of Neurosurgery, University of Alberta, Edmonton, AB Canada; 16 Departments of Orthopedics and Neurosurgery, University of Manitoba, Winnipeg, Canada; 17 Combined Neurosurgical and Orthopedic Spine Program, Department of Orthopaedic Surgery, University of British Columbia, Vancouver, BC Canada; 18 Division of Neurosurgery, Department of Surgery, University of British Columbia, Vancouver, BC Canada

**Keywords:** Orthopaedics, Outcomes research, Musculoskeletal system, Neurological disorders, Disability

## Abstract

This retrospective study of prospectively collected data aimed to identify unique pain and disability trajectories in patients following lumbar discectomy surgery. Patients of this study population presented chiefly with lumbar radiculopathy and underwent discectomy surgery from thirteen sites enrolled in the CSORN registry. Outcome variables of interest included numeric rating scales for leg/back pain and modified Oswestry disability index scores at baseline, 3, 12, and 24 months post-operatively. Latent class growth analysis was used to identify distinct courses in each outcome. Data from 524 patients revealed three unique trajectories for leg pain (excellent = 18.4%, good = 55.4%, poor = 26.3%), disability (excellent = 59.7%, fair = 35.6%, poor = 4.7%) and back pain (excellent = 13.0%, good = 56.4%, poor = 30.6%). Construct validity was supported by statistically significant differences in the proportions of patients attaining the criteria for minimal important change (MIC; 30%) or clinical success in disability (50% or Oswestry score ≤ 22) (p < 0.001). The variable proportions of patients belonging to poor outcome trajectories shows a disconnect between improved disability and persistence of pain. It will be beneficial to incorporate this information into the realm of patient expectation setting in concert with future findings of potential factors predictive of subgroup membership.

## Introduction

Commonly referred to as sciatica, lumbosacral radiculopathy is a symptom of spine pathology characterized by pain from the back/buttock radiating into the lower extremity and may also include motor weakness and sensory disturbances^1^. In most cases, lumbar radiculopathy is caused by compression of nerve roots from disease in the intervertebral disc or associated structures^1^. The point prevalence of lumbosacral radiculopathy ranges from 1.6 to 13.4% in men and women between 45 and 64 years of age^2^.

Lumbar discectomy is a common surgical procedure to treat lumbosacral radiculopathy due to disc pathology^3^. Current evidence shows that lumbar discectomy is superior to non-surgical treatment for pain relief and improvement in function in the short-term, but not at mid-term and long-term follow-up^4,5^. Whether approached using a minimally invasive or open technique, lumbar discectomy surgery is supported as a highly effective procedure for the majority of patients, though with variability in outcome^6–11^. For instance, studies have reported that between 30 and 70% of patients indicate pain as a continuing problem following surgery, suggesting that many patients do not experience a favorable postoperative outcome trajectory^12,13^. Research in other surgical spine interventions such as decompression with or without fusion for spinal stenosis supports that different pain and disability trajectories may exist within a broader patient population undergoing lumbosacral surgery^14^.

Therefore, we aim to explore the distinct courses of pain and disability following discectomy surgery in patients affected by lower extremity radiculopathy. Specifically, the objectives of this study are to: (1) identify the postoperative trajectories of pain and disability in patients who underwent lumbar discectomy surgery, and (2) investigate the construct validity of the trajectory subgroups based on clinically meaningful changes in pain and disability. These results may be useful in understanding prognosis and postoperative course of symptoms experienced by patients.

## Materials and methods

### Study design and participants

This was a retrospective longitudinal analysis of prospective data collected from patients enrolled in the Canadian Spine Outcomes and Research Network (CSORN) from January 2015 to June 2020. The CSORN is a national collaboration of orthopaedic and neurological spine surgeons that maintains a registry of spine surgery outcomes. Patient-reported data were collected at pre-operative baseline, and 3, 12, and 24 months after surgery.

Inclusion criteria were as follows: (1) age ≥ 18 years (or provincial age of majority), (2) lumbar radiculopathy as diagnosed by the attending surgeon, and (3) received open or minimally invasive lumbar discectomy surgery with a posterior midline or paracentral approach for degenerative pathology. We excluded patients who underwent surgery other than discectomy (e.g., fusion) and those with non-degenerative pathology (e.g., fracture, tumour, infection). Ethical approval for this study was provided by the Horizon Health Network Research Ethics Boards (2020-2869; file no. 100828). Patients were provided study information and written informed consent was obtained before study enrolment by signing forms approved by the Horizon Health Network Research Ethics Boards (file no. 10269). All methods were performed in accordance with the principles of the ICH Harmonized Tripartite Guidelines: Good Clinical Practice and the Tri-Council Policy Statement.

### Measures of interest

Prior to surgery, baseline demographic and clinical data were collected from the patient and treating surgeon (Table 1). These variables consisted of age, sex, body mass index, smoking status, previous spine surgery, time with condition, number of comorbidities and number of levels operated on. Continuous variables are presented as means ± standard deviation (SD) while categorical variables are broken down into counts and percentages. The main outcome measures of interest were leg pain intensity, back pain intensity, and pain-related disability.

**Table 1:** Baseline demographics and surgical details of the eligible patient sample.

Variable	Patient sample
Age [mean ± SD] (N = 524)	47.5 ± 14.3
**Sex (N = 524)**	
Female	256 (48.9%)
Body mass index [mean ± SD] (N = 501)	27.8 ± 5.5
Missing	23 (4.4%)
**Smoking status (N = 515)**	
Non-smoker	412 (80.0%)
Current smoker	103 (20.0%)
**Previous spine surgery (N = 522)**	
Yes	55 (10.5%)
No	462 (88.5%)
Missing	5 (1.0%)
**Time with condition (N = 522)**	
> 6 weeks	6 (1.2%)
6–12 weeks	32 (6.1%)
3–6 months	84 (16.1%)
6–12 months	125 (23.9%)
12–24 months	77 (14.7%)
Greater than 24 months	195 (37.4%)
Missing	3 (0.6%)
**Number of comorbidities (N = 520)**	
0	85 (16.3%)
1	125 (24.0%)
2	146 (28.1%)
3	77 (14.8%)
4	49 (9.4%)
> 4	38 (7.3%)
**Number of levels operated on (N = 523)**	
1	496 (94.8%)
2	23 (4.4%)
3	3 (0.6%)
> 3	1 (0.2%)

#### Leg pain and back pain

The 11-point numeric pain rating scale (NRS) was used to separately quantify leg and back pain intensity^15^. Scores provided a measure of average pain intensity over the previous 24 h period where scores could range from 0 (no pain) to 10 (worst pain imaginable) and were categorized as: 0 ‘no pain’, 1–3 ‘mild pain’, 4–6 ‘moderate pain’, and 7–10 ‘severe pain’^16,17^. The NRS has good test–retest reliability and responsiveness^18,19^. The level of minimal important change (MIC) in pain intensity is estimated to be 30%^20^.

#### Pain-related disability

The modified Oswestry Disability Index (ODI) was used to measure pain-related disability^21^. The questionnaire evaluated difficulty performing 10 functional activities with an overall score that ranged from 0 to 100 (higher scores represented greater disability). The categorization of total scores was: 0–20 ‘minimal disability’, 21–40 ‘moderate disability’, 41–60 ‘severe disability’, 61–80 ‘crippled’, and 81–100 ‘bedbound or exaggerating’^22^. The questionnaire has excellent test–retest reliability and responsiveness^23^. The MIC is estimated to be 30%^20^. We categorized patients as meeting criteria for MIC, as well as, criteria for relative (50% improvement)^14^ and absolute (ODI score ≤ 22)^24^ estimates of clinical success.

### Data analysis

All analyses were conducted using Stata 16.1 software (StataCorp, College Station, TX, USA). To identify the trajectories of pain and disability in patients who underwent lumbar discectomy (aim one), we constructed separate latent class growth analysis models for each study outcome. Compared to variable-centred models that investigate associations between variables (e.g., regression), person-centred approaches such as latent class growth analysis seek to identify subgroups of individuals who share particular attributes (e.g., the course of pain or disability over time)^25^. In this way, person-centred models can identify meaningful patient subgroups based on the development of their symptoms over time, and not just at a particular time point, which better aligns with the perspectives of patients and clinicians^26^.

Patients who undergo lumbar discectomy for radiculopathy may primarily experience axial or peripheral symptoms with varying levels of disability. For example, some patients report high levels of leg pain intensity with minimal back pain, while others report predominant back pain. Moreover, some patients have minimal pain but are identified as surgical candidates because of neurological impairment. Given these heterogeneous presentations and the nature of the study outcomes, we excluded patients with low baseline scores on the model outcome: NRS scores < 3 for the leg pain and back pain models, and ODI score < 21 for the disability model. These classifications were specific to the model outcome, and therefore, patients could have contributed data to one, two, or all three trajectory models. We also excluded patients with missing outcome data at the preoperative baseline and those with outcome data at less than two time points. Latent class growth analysis handles missing data with maximum likelihood estimation, resulting in asymptotically unbiased parameter estimates when data are missing at random^26^.

Starting with single class models and a censored normal distribution, we increased the number of classes and complexity of polynomial distributions until optimal models were identified. Initial decisions regarding optimal model specification were made using the Bayesian information criteria. Final model selection was based on additional statistical and clinical judgments. We tested the models using four a priori diagnostic criteria: (1) a minimum average posterior probability of group membership of 0.7; (2) close correspondence between the estimated group membership probability and the proportion of participants assigned to each group based on the posterior probability; (3) minimum odds of correct classification greater than five; and (4) precise confidence intervals around estimations of group membership probabilities^26,27^. The subgroups identified by the model were assigned descriptive labels based on clinical judgement and the proportion of patients meeting benchmarks for clinically important improvement and clinical success.

To investigate the construct validity of the trajectory subgroups (aim two), we generated descriptive statistics for leg pain, back pain, and pain-related disability, stratified by trajectory class and calculated the proportion of patients within each trajectory who met clinical benchmarks for minimal improvement and clinical success at the 12-month follow-up. The measurement of MIC as a 30% change from baseline was selected over other available approaches to allow for the application of a consistent, simplified standard for both pain and disability outcomes^20^. Differences between trajectory classes were examined with Fisher’s exact test. Alpha was 5% for all inferential analyses.

## Results

**Figure 1:**
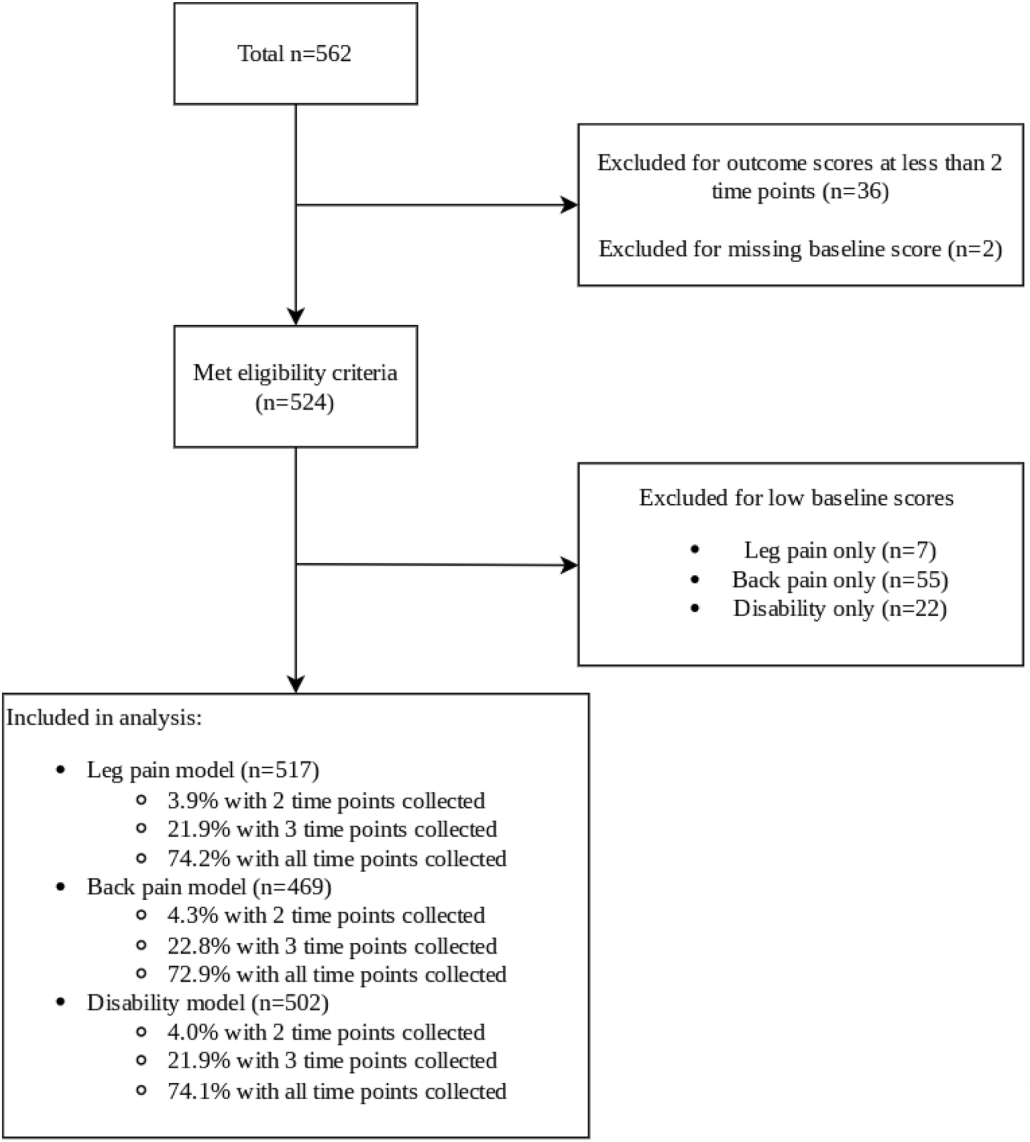
Study flow diagram.

Of 562 patients with lumbar radiculopathy who underwent discectomy surgery, 36 patients (6.4%) were excluded for having data at less than two time points and two (0.4%) were excluded for missing baseline data. In total, 524 patients treated by one of 57 surgeons from 13 institutions contributed data to one or more trajectory models. Data from 7 patients in the leg pain model, 55 patients in the back pain model, and 22 patients in the disability model were excluded owing to low baseline scores representing minimal pain (NRS < 3) or disability (ODI < 21; Fig. 1). Table 1 reports the descriptive data for all included patients. A list of all patient comorbidities and additional descriptive data stratified by trajectory subgroup and can be found as Supplementary Tables S1 and S2a–c, respectively.

Final models consisted of 3-class solutions that met all prespecified model diagnostics (Table 2). Table 3 presents the average pain and disability outcomes across all time points, stratified by trajectory subgroup. The proportions of patients achieving different clinical outcome criteria within each trajectory subgroup are summarized in Table 4. The between subgroup differences were all found to be significant for the various measures of MIC and clinical success (p < 0.001).

**Table 2:** Assignment to trajectory groups based on outcomes of leg pain, back pain, and disability.

	Average posterior probability^a^	Estimated membership (95% CI)	Assigned membership (%)	Odds of correct classification^b^
**Leg pain trajectory groups (N = 517)**				
1, 'excellent'	0.85	18.4 (12.1–24.7) %	19.1	22.95
2, 'good'	0.92	55.4 (49.0–61.8) %	54.4	9.12
3, 'poor'	0.90	26.3 (21.2–31.4) %	26.5	25.23
**Back pain trajectory groups (N = 469)**				
1, 'excellent'	0.84	13.0 (5.3–20.7) %	12.2	37.19
2, 'good'	0.91	56.4 (49.0–63.8) %	56.5	7.47
3, 'poor'	0.90	30.6 (24.9–36.4) %	31.3	19.27
**Disability trajectory groups (N = 502)**				
1, 'excellent'	0.96	59.7 (54.6–64.8) %	60.6	14.54
2, 'fair'	0.93	35.6 (30.6–40.5) %	35.1	23.66
3, 'poor'	0.90	4.7 (2.1–7.4) %	4.4	194.6

^a^Minimum threshold = 0.70.

^b^Minimum threshold = 5.0.

**Table 3:** Scores for clinical outcomes according to trajectory group across time.

	Preoperative	3 months	12 months	24 months
**Leg pain trajectory groups (0–10 leg NRS score)**				
1, 'excellent'	7.5 ± 1.8	0.8 ± 1.2	0.2 ± 0.4	0.0 ± 0.0
2, 'good'	7.5 ± 1.7	2.3 ± 1.9	2.3 ± 1.7	2.3 ± 2.0
3, 'poor'	8.1 ± 1.6	6.1 ± 2.3	6.4 ± 2.1	6.4 ± 2.2
**Back pain trajectory groups (0–10 back NRS score)**				
1, 'excellent'	6.2 ± 2.4	0.8 ± 1.1	0.3 ± 0.6	0.1 ± 0.4
2, 'good'	6.5 ± 1.9	2.6 ± 1.6	2.4 ± 1.5	2.4 ± 1.6
3, 'poor'	7.6 ± 1.6	5.5 ± 2.0	6.4 ± 1.9	6.2 ± 1.8
**Disability trajectory groups (0–100 ODI score)**				
1, 'excellent'	46.3 ± 13.6	16.5 ± 12.4	10.3 ± 9.6	9.6 ± 9.1
2, 'fair'	55.1 ± 12.9	39.9 ± 12.6	38.3 ± 12.1	37.8 ± 11.3
3, 'poor'	68.4 ± 10.2	64.1 ± 12.1	63.1 ± 15.0	66.8 ± 9.9

Values are mean ± SD.

*NRS* numeric rating scale, *ODI* modified Oswestry disability index.

**Table 4:** Breakdown of proportions of patients achieving clinical criteria at 12 month follow-up.

Groups	Leg pain MIC^a^ (%)	Back pain MIC^b^ (%)	ODI MIC^c^ (%)	Relative ODI success^d^ (%)	Absolute ODI success^e^ (%)
**Leg pain trajectory groups**					
1, 'excellent'	100	88.1	96.5	90.6	91.8
2, 'good'	88.0	78.3	81.5	64.8	62.5
3, 'poor'	35.2	38.0	43.9	22.4	18.2
**Back pain trajectory groups**					
1, 'excellent'	97.9	97.9	97.9	97.9	95.8
2, 'good'	86.7	90.1	86.0	68.7	68.1
3, 'poor'	50.0	26.8	39.6	17.1	10.5
**Disability trajectory groups**					
1, 'excellent'	87.5	86.8	92.1	85.3	85.4
2, 'fair'	62.6	46.0	51.1	19.4	9.3
3, 'poor'	23.5	11.1	17.7	5.9	0.0

Statistical significance (*p* < 0.001) was obtained for the between-group differences in the proportion of patients meeting each of the clinical criteria.

Green ≥ 75%; yellow 50–74%; red < 50%.

*MIC* minimal important change, *ODI* modified Oswestry disability index.

^a^≥ 30% reduction in NRS for leg pain.

^b^≥ 30% reduction in NRS for back pain.

^c^≥ 30% reduction in ODI.

^d^≥ 50% reduction in ODI.

^e^ODI score ≤ 22.

### Leg pain trajectories

**Figure 2:**
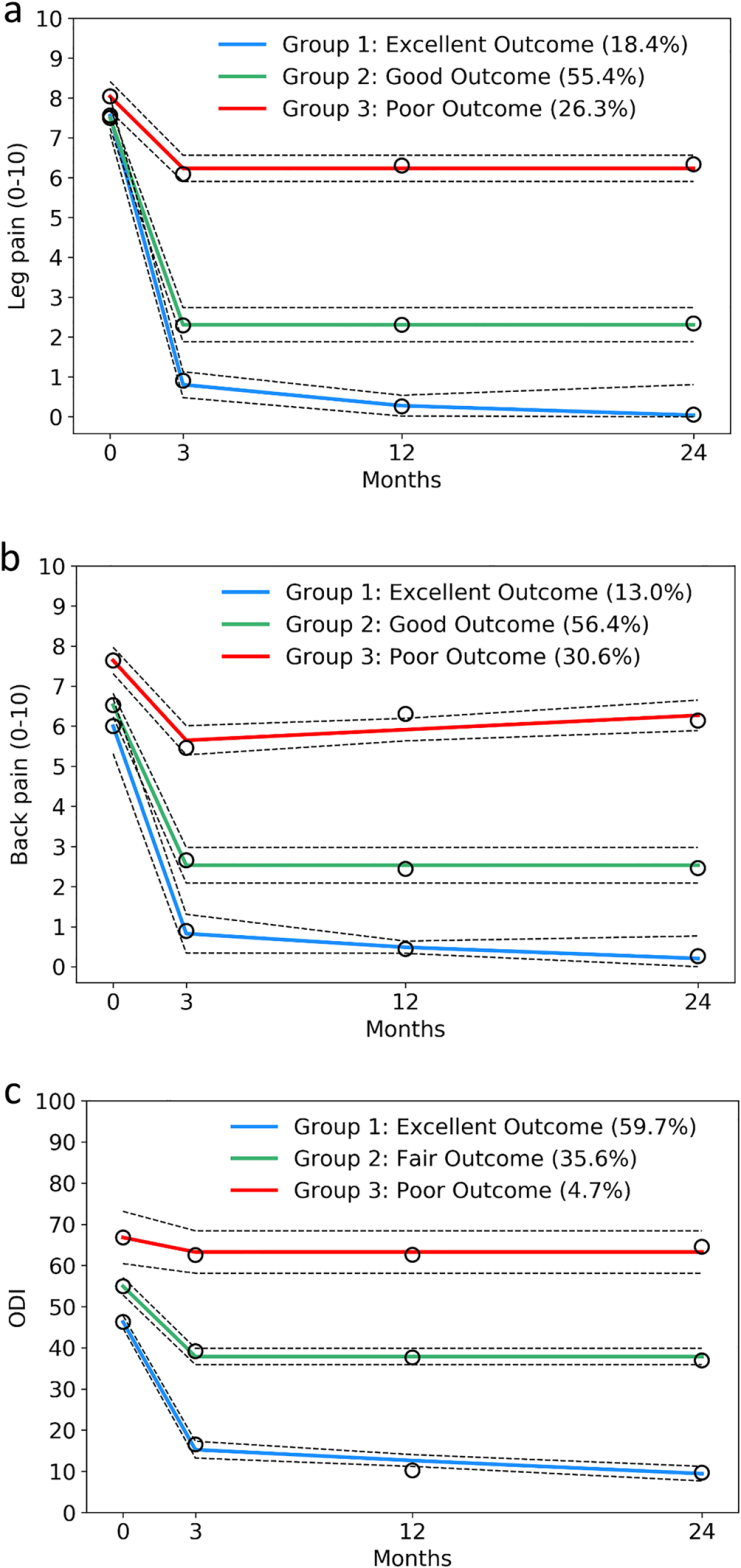
3-Class clinical outcome trajectory groups. (**a**) Leg pain trajectories (N = 517); (**b**) back pain trajectories (N = 469); (**c**) disability trajectories (N = 502). Point estimates represent the mean score at each time point (0–10 numeric pain rating scale or 0–100 modified Oswestry disability index). Dotted lines represent 95% confidence intervals.

The distinct trajectory subgroups for the leg pain model are presented in Fig. 2a. Group 1 (excellent outcome) consisted of the 18.4% of patients who experienced substantial improvement in leg pain intensity, dropping from a mean score (± SD) of 7.5 ± 1.8 points to 0.8 ± 1.2 points on the NRS at 3 months and remaining stable thereafter. MIC was achieved by all patients in Group 1 and nearly all (90.6–91.8%) experienced a successful outcome at 12 months. Group 2 (good outcome) comprised 55.4% of patients who underwent a recovery pattern marked by a large drop in leg pain from baseline to 3 months and then maintained a persistent level of leg pain in the upper range of the mild category (mean ± SD of 2.3 ± 1.9 points on NRS) up until 24 months. Relative and absolute success in disability at 12 months were achieved by 62.5–64.8% of this group. The remaining 26.3% of patients were categorized in Group 3 (poor outcome). The mean baseline score for this group was similar to that of the other two groups yet a small level of improvement was seen from baseline to 3 months which remained stable up to 24 months follow-up. MIC in leg pain was present in just 35.2% of patients in Group 3, and 18.2% to 22.4% of these patients reported successful relative and absolute disability outcomes at the 12 month follow-up.

### Back pain trajectories

Figure 2b displays the trajectory subgroups identified for the back pain model. Group 1 (excellent outcome) included 13.0% of patients whose back pain intensity dropped substantially into the range of minimal-to-no pain at 3 months (reduction from a mean score ± SD of 6.2 ± 2.4 points to 0.8 ± 1.1 points). Nearly all patients in Group 1 achieved a successful outcome (95.8% to 97.9%) while all patients reported MIC in back pain. The 56.4% of patients in Group 2 (good outcome) experienced moderate improvement in their back pain that remained in the upper range of the mild category from 3 to 24 months follow-up (average reduction from a mean score ± SD of 6.5 ± 1.9 points to 2.6 ± 1.6 points). Success rates of 68.7–68.1% were seen for patients in this group. Group 3 (poor outcome) comprised 30.6% of patients who reported minimal or no improvement in pain at the 3, 12 and 24 month follow-ups. Only a small subset of patients in this group (10.5–17.1%) attained an outcome of success 12 months post-surgery.

### Pain-related disability

Trajectory subgroups identified by the disability model are presented in Fig. 2c. Patients in Group 1 (excellent outcome) predominantly experienced MIC in leg pain (87.5%) and back pain (86.8%) along with relative and absolute disability success (85.3–85.4%) at 12 months. The 35.6% of patients in Group 2 (fair outcome) reported moderate improvements in disability. While only 9.3–19.4% reached the absolute and relative measures of success, it is of note that about half of this group reached MIC at 12 months. Group 3 (poor outcome) included just 4.7% of patients who saw very minimal reduction in their disability postoperatively such that they continued to experience severe disability. In this group, 23.5% achieved MIC in disability and up to 5.9% attained a successful outcome at 12 months.

## Discussion

This study provides evidence of the different postoperative pain and disability trajectories experienced by patients with lumbar radiculopathy who undergo discectomy surgery. While poor disability outcomes were experienced by few patients, 26.3–30.6% of patients reported persistence of their back or leg pain post-surgery. Evidence supporting construct validity of each trajectory subgroup was identified by comparing the proportions of patients who met chosen thresholds for minimum clinically-important change and clinical success.

Prior available evidence suggests that lumbar discectomy yields a high yet variable success rate, ranging from 76 to 90% based on numerous measurement parameters^6–11^. Given these available findings, this study explored lumbar discectomy as the surgery type of interest to better understand how this variability may be attributed to distinct trajectory membership. Rather than centering the investigation on a single outcome measure or an absolute score cut-off in follow-up, the current investigation further provides a breakdown using multiple criteria for clinically important change and clinical success.

Where the existing literature generally explores the outcomes of patients following discectomy surgery by describing average changes for the population as a whole, the aim in this investigation was to delve deeper into the different classes of recovery patterns that may exist for the outcome measures of pain and disability across time. A recent systematic review and meta-analysis reported the leg pain experienced by patients following discectomy surgery to undergo a sharp decrease that is ultimately preserved in the long-term^28^. The current study found 73.8% of patients fell into one of two outcome trajectories resembling this type of recovery pattern with the remaining quarter of patients instead reporting minimal change and maintaining a high level of leg pain after 24 months. The subgroups identified for back pain were similar to those of leg pain both in class membership and trajectory, although more patients belonged to the poor subgroup compared to the excellent subgroup for this outcome measure. While a synthesis of results for back pain in the literature describes notable improvement that is preserved over time, the subgroups provide evidence of a considerable 30.6% of patients with a much less beneficial recovery^28^. Unlike the pain subgroups, those for disability indicated that nearly the entire patient population (95.3%) experienced an excellent-fair pattern of recovery. Other investigations have also found substantial reductions in mean disability when averaging the population as a whole^8,28,29^.

Interestingly, leg pain improvement appeared to be independent of baseline leg pain as the three distinct subgroups reported very similar preoperative pain levels. For the back pain outcome, however, the mean baseline pain level for the poor subgroup fell within the severe range which was not the case for the other two subgroups. The 4.7% of patients who did not achieve disability improvement also belonged to the severe disability category at baseline unlike the remainder of the population.

It is important to note that the novel subgroups identified for patients experiencing radiculopathy who underwent discectomy differ greatly from those described in a recent study similarly centered on the trajectory subgroups for patients receiving decompression to treat lumbar spinal stenosis^14^. This comparison supports the notion that distinct groupings of recovery patterns exist which vary according to the clinical features of the overall population of interest. The subgroups identified in this study, particularly the disparity in belonging to the poor outcome group seen between pain and disability, provide valuable knowledge of relevance to the shared-decision making process. To contribute further insight to the selection and decision-making steps, a research project is presently underway with the objective of isolating preoperative factors predictive of membership to one subgroup over another.

This study established different trajectories for pain and disability while identifying evidence for construct validity using a large set of patient data collected on a national scale. The three follow-up time points available allowed for subgroups of recovery patterns to be modelled both in the short- and long-term. Utilizing a trajectory technique enabled an understanding of unique subgroups of patient outcomes that would be otherwise unexplored when referring to average scores for the population as a whole.

A recognized limitation of this investigation is the retrospective nature in which it was conducted which leaves the potential for uncontrolled confounding factors to influence the results. Though the vast majority of patients included in the study had data at preoperative baseline and at least 2 of 3 follow-up time points, non-randomly missing data may introduce bias as latent class growth analysis assumes data to be missing at random. However, complete data were available for approximately three-quarters of the patient population, thus missing data is unlikely to have a significant impact on the study results.

## Conclusion

Patients with lumbar radiculopathy experience unique pain and disability trajectories after discectomy. These findings suggest that few patients with these clinical features demonstrate poor outcomes at the level of disability which is not similarly seen in the proportion of patients reporting poor outcomes for postoperative pain. This information is of importance to the realm of patient expectation setting while taking into consideration the principal goals expressed for recovery.

## Supplementary Information

Supplementary Tables.

## Data Availability

The datasets generated during and/or analysed during the current study are not publicly available due to legal and ethical restrictions but are available from the Canadian Spine Outcomes and Research Network (contact Greg McIntosh via gmcintosh@spinecanada.ca) for researchers who meet the criteria for access to confidential data.
