# Supplementary material for: Postoperative recovery patterns following discectomy surgery in patients with lumbar radiculopathy
Source: Sci Rep. 2022 Jul 1;12:11146. doi: 10.1038/s41598-022-15169-8 (PMC9249755; doi:10.1038/s41598-022-15169-8)
Supplement: Supplementary file 1 — Supplementary Tables. [file 41598_2022_15169_MOESM1_ESM.pdf]

Full Title: Postoperative recovery patterns following discectomy surgery in patients with lumbar radiculopathy

Short Title: Discectomy surgery recovery patterns

Shuaijin Wang[1], Jeffrey Hebert[2*], Edward Abraham[1,3,4,5], Amanda Vandewint[3,5], Erin Bigney[3,5], Eden Richardson[3,5,8], Dana El-Mughayyar[2,3,5], Najmedden Attabib[1,3,5], Niels Wedderkopp[6,7], Stephen Kingwell[9], Alex Soroceanu[10], MH Weber[11], Hamilton Hall[12], Joel Finkelstein[13], Christopher S Bailey[14], Kenneth Thomas[10], Andrew Nataraj[15], Jerome Paquet[11], Michael G Johnson[16], Charles Fisher[17], Y Raja Rampersaud[18], Nicolas Dea[19], Chris Small[1,3,4,5], Neil Manson[1,3,4,5]

1 Faculty of Medicine, Dalhousie Medicine New Brunswick, Saint John, New Brunswick, Canada
2 Faculty of Kinesiology, University of New Brunswick, Fredericton, New Brunswick, Canada
3 Canada East Spine Centre, Saint John, New Brunswick, Canada
4 Saint John Orthopaedics, Saint John, New Brunswick, Canada
5 Horizon Health Network, Saint John, New Brunswick, Canada
6 Department of Regional Health Research, University of Southern Denmark, Odense, Denmark
7 The Orthopedic Department, Hospital of Southwestern Jutland, Esbjerg, Denmark
8 Canadian Spine Outcomes and Research Network, Markdale, Ontario, Canada
9 The Ottawa Hospital-Civic Campus, Ottawa, Ontario, Canada
10 Department of Surgery, University of Calgary, Calgary, Alberta, Canada
11 McGill University, Montreal, Quebec, Canada
12 Department of Surgery, University of Toronto, Toronto, Ontario, Canada
13 Division of Orthopedics and Spine Surgery, Sunnybrook Health Sciences Centre, Toronto, Ontario, Canada
14 London Health Science Centre, Western University, London, Ontario, Canada
15 Department of Surgery, Division of Neurosurgery, University of Alberta, Edmonton, Alberta, Canada
16 Departments of Orthopedics and Neurosurgery, University of Manitoba, Winnipeg, Canada
17 Combined Neurosurgical and Orthopedic Spine Program, Department of Orthopaedic Surgery, University of British Columbia, Vancouver, British Columbia, Canada
18 Department of Surgery, University of Toronto, Toronto, Ontario, Canada
19 Division of Neurosurgery, Department of Surgery, University of British Columbia, Vancouver, British Columbia, Canada

*Corresponding author:
Email: j.hebert@unb.ca (JH)

Supplementary Table S1. Comorbid conditions reported in the study population.

**Comorbidities**	
Total No.	524
Anemia	41 (7.8)
Cancer	38 (7.3)
Cardiovascular disease<br>Hypertension<br>Heart attack in last 6 months<br>Congestive heart failure	 111 (21.2)<br>8 (1.5)<br>6 (1.1)
Chronic pain<br>Back/neck pain<br>Frequent headache	 334 (63.7)<br>96 (18.3)
Chronic respiratory disease<br>Asthma<br>Chronic pulmonary disease or current pneumonia	 71 (13.5)<br>11 (2.1)
Depression	136 (30)
Dementia	1 (0.2)
HIV	1 (0.2)
Kidney Disease	4 (0.8)
Liver Disease	 7 (1.3)<br>1 (0.2)
Metabolic Disease<br>Diabetes<br>High cholesterol	 35 (6.7)<br>67 (12.8)
Osteoarthritis	59 (11.3)
Ulcer Disease (stomach or peptic ulcers)	23 (4.4)
Vascular disease	11 (2.1)

Supplementary Table S2a. Leg pain subgroups: baseline demographics and surgical details of the eligible patient sample.

**Variable**	**"Excellent" (N = 99)**	**"Good" (N = 281)**	**"Poor" (N = 137)**
**Age [mean ± SD] (N = 517)**	46.5 ± 15.1	46.3 ± 13.7	50.2 ± 14.3
**Female**	53 (53.5%)	117 (41.6%)	84 (61.3%)
**Body Mass Index [mean ± SD] (N = 503)**<br>Missing = 9 (1.8%)	27.1 ± 6.4	27.6 ± 5.2	28.9 ± 5.6
**Smoking Status (N = 508)**			
Non-smoker<br>Current smoker<br>Missing = 9 (1.8%)	79 (81.4%)<br>18 (18.6%)	226 (81.6%<br>51 (18.4%)	100 (74.6%)<br>34 (25.4%)
**Previous Spine Surgery (N = 515)**			
Yes<br>No	4 (4.0%)<br>94 (95.0%)	29 (10.4%)<br>249 (88.9%)	21 (15.4%)<br>113 (83.1%)

Missing	1 (1.0%)	2 (0.7%)	2 (1.5%)
**Time with Condition (N = 515)**			
< 6 weeks<br>6 to 12 weeks<br>3 to 6 months<br>6 to 12 months<br>12 to 24 months<br>> 24 months<br>Unknown	0<br>6 (6.1%)<br>12 (12.1%)<br>21 (21.2%)<br>14 (14.1%)<br>45 (45.5%)<br>1 (1.0%)	2 (0.7%)<br>21 (7.5%)<br>46 (16.4%)<br>67 (23.9%)<br>45 (16.1%)<br>98 (35.0%)<br>1 (0.4%)	4 (2.9%)<br>5 (3.7%)<br>24 (17.7%)<br>33 (24.3%)<br>18 (13.2%)<br>51 (37.5%)<br>1 (0.7%)
**Number of Comorbidities (N = 513)**			
0<br>1<br>2<br>3<br>>3	23 (23.5%)<br>29 (29.6%)<br>30 (30.6%)<br>9 (9.2%)<br>7 (7.1%)	49 (17.6%)<br>74 (26.5%)<br>76 (27.2%)<br>38 (13.6%)<br>42 (15.1%)	12 (8.8%)<br>20 (14.7%)<br>38 (28.0%)<br>29 (21.3%)<br>37 (27.2%)
**Number of Levels Operated On (N = 516)**			
1<br>2<br>3<br>>3	94 (95.0%)<br>5 (5.0%)	266 (95.0%)<br>12 (4.3%)<br>2 (0.7%)	129 (94.2%)<br>6 (4.4%)<br>1 (0.7%)<br>1 (0.7%)

Supplementary Table S2b. Back pain subgroups: baseline demographics and surgical details of the eligible patient sample.

Variable	"Excellent" (N = 57)	"Good" (N = 265)	"Poor" (N = 147)
**Age [mean ± SD] (N = 469)**	51.4 ± 15.4	45.4 ± 14.0	49.4 ± 14.3
**Female**	25 (43.9%)	124 (46.8%)	82 (55.8%)
**Body Mass Index [mean ± SD] (N = 447)**<br>Missing = 56 (11.1%)	27.0 ± 4.6	27.4 ± 5.5	29.3 ± 5.9
**Smoking Status (N = 460)**			
Non-smoker<br>Current smoker<br>Missing = 57 (12.4%)	42 (76.4%)<br>13 (23.6%)	214 (82.0%)<br>47 (18.0%)	104 (72.2%)<br>40 (27.8%)
**Previous Spine Surgery (N = 467)**			
Yes<br>No<br>Missing	2 (3.5%)<br>54 (94.7%)<br>1 (1.8%)	27 (10.2%)<br>234 (88.6%)<br>3 (1.1%)	23 (15.8%)<br>122 (83.65%)<br>1 (0.7%)
**Time with Condition (N = 467)**			
< 6 weeks<br>6 to 12 weeks<br>3 to 6 months<br>6 to 12 months<br>12 to 24 months	1 (1.7%)<br>4 (7.0%)<br>14 (24.6%)<br>12 (21.1%)<br>5 (8.8%)	1 (0.4%)<br>15 (5.7%)<br>38 (14.4%)<br>68 (25.7%)<br>46 (17.4%)	4 (2.7%)<br>7 (4.8%)<br>20 (13.7%)<br>32 (21.9%)<br>20 (13.7%)

> 24 months<br>Unknown	21 (36.8%)<br>0	94 (35.6%)<br>2 (0.8%)	62 (42.5%)<br>1 (0.7%)
**Number of Comorbidities (N = 466)**			
0<br>1<br>2<br>3<br>>3	13 (22.8%)<br>18 (31.6%)<br>16 (28.1%)<br>4 (7.0%)<br>6 (10.5%)	42 (15.9%)<br>72 (27.3%)<br>76 (28.8%)<br>38 (14.4%)<br>36 (13.6%)	14 (9.7%)<br>20 (13.8%)<br>38 (26.2%)<br>30 (20.7%)<br>43 (29.6%)
**Number of Levels Operated On (N = 468)**			
1<br>2<br>3<br>>3	54 (94.8%)<br>2 (3.5%)<br>1 (1.7%)	254 (95.8%)<br>10 (3.8%)<br>1 (0.4%)	135 (92.5%)<br>9 (6.1%)<br>1 (0.7%)<br>1 (0.7%)

Supplementary Table S2c. Disability subgroups: baseline demographics and surgical details of the eligible patient sample.

Variable	"Excellent" (N = 304)	"Good" (N = 176)	"Poor" (N = 22)
**Age [mean ± SD] (N = 502)**	46.6 ± 14.1	49.6 ± 14.4	48.9 ± 13.1
**Female**	137 (45.1%)	96 (54.6 %)	14 (63.6%)
**Body Mass Index [mean ± SD] (N = 481)**<br>Missing = 22 (4.4%)	27.5 ± 5.3	28.6 ± 5.9	29.2 ± 5.6
**Smoking Status (N = 493)**			
Non-smoker<br>Current smoker<br>Missing = 24 (4.9%)	246 (82.5%)<br>52 (17.5%)	135 (77.6%)<br>39 (22.4%)	13 (61.9%)<br>8 (38.1%)
**Previous Spine Surgery (N = 500)**			
Yes<br>No<br>Missing	26 (8.6%)<br>274 (90.4%)<br>3 (1.0%)	20 (11.4%)<br>156 (88.6%)<br>0	6 (28.6%)<br>14 (66.7%)<br>1 (4.7%)
**Time with Condition (N = 500)**			
< 6 weeks<br>6 to 12 weeks<br>3 to 6 months<br>6 to 12 months<br>12 to 24 months<br>> 24 months<br>Unknown	3 (1.0%)<br>21 (6.9%)<br>49 (16.2%)<br>83 (27.4%)<br>44 (14.5%)<br>101 (33.3%)<br>2 (0.7%)	2 (1.1%)<br>10 (5.7%)<br>31 (17.6%)<br>37 (21.0%)<br>27 (15.4%)<br>69 (39.2%)<br>0	1 (4.8%)<br>1 (4.8%)<br>3 (14.3%)<br>3 (14.3%)<br>3 (14.3%)<br>9 (42.9%)<br>1 (4.8%)
**Number of Comorbidities (N = 499)**			
0<br>1<br>2	59 (19.5%)<br>90 (29.8%)<br>79 (26.2%)	17 (9.7%)<br>26 (14.8%)<br>55 (31.2%)	3 (14.3%)<br>2 (9.5%)<br>6 (28.6%)

3<br>>3	39 (12.9%)<br>35 (11.6%)	35 (19.9%)<br>43 (24.4%)	3 (14.3%)<br>7 (33.3%)
**Number of Levels Operated On (N = 503)**			
1<br>2<br>3<br>>3	289 (95.1%)<br>13 (4.3%)<br>2 (0.6%)	165 (94.3%)<br>9 (5.1%)<br>1 (0.6%)	20 (90.9%)<br>1 (4.5%)<br>1 (4.5%)
